# Corticosteroid reduction by addition of cetirizine and montelukast in biopsy-proven minimal-change nephrotic syndrome concomitant with allergic disorders

**DOI:** 10.1038/s41598-020-58463-z

**Published:** 2020-01-30

**Authors:** Yoichi Oshima, Keiichi Sumida, Masayuki Yamanouchi, Noriko Hayami, Akinari Sekine, Hiroki Mizuno, Masahiro Kawada, Rikako Hiramatsu, Eiko Hasegawa, Tatsuya Suwabe, Junichi Hoshino, Naoki Sawa, Takeshi Fujii, Kenmei Takaichi, Yoshifumi Ubara

**Affiliations:** 1 Nephrology Center, Toranomon Hospital, Tokyo, Japan; 2 Department of Nephrology, Endocrinology, and Metabolism, Keio University School of Medicine, Tokyo, Japan; 3 Division of Nephrology, Department of Medicine, University of Tennessee Health Science Center, Memphis, TN USA; 4 Department of Pathology, Toranomon Hospital, Tokyo, Japan; 5 Okinaka Memorial Institute for Medical Research, Toranomon Hospital, Tokyo, Japan

**Keywords:** Minimal change disease, Risk factors, Comorbidities

## Abstract

Recent reports suggest helper T-cell abnormalities in minimal-change nephrotic syndrome (MCNS), which often complicate allergic disorders that show a similar helper T-cell profile with Th2/Th17 predominance. However, the effect of anti-allergy therapy on MCNS remains unknown. This retrospective study included 51 patients with biopsy-proven MCNS recruited between November 2012 and October 2015, with follow-up through November 2017. We analyzed relapse and temporal daily corticosteroid dose with and without co-administration of histamine H1 receptor antagonist, cetirizine, and cysteinyl-leukotriene receptor antagonist, montelukast, as well as between baseline and after follow-up. Thirteen patients were treated with cetirizine and montelukast in addition to conventional therapy, whereas 38 patients were treated by conventional therapy only, consisting of corticosteroids and immunosuppressants. To adjust for baseline clinical characteristics, a 1:1 propensity score–matched model was applied. The clinical characteristics of the two groups after matching were similar at baseline. The treatment group showed a significant reduction in the lowest daily dose of oral prednisolone throughout the entire treatment course after the study compared to that of baseline (p < 0.025), which was not observed in the control group (p = 0.37), and showed significantly higher percentage of patients establishing corticosteroid-free state for the first time throughout the entire treatment course by addition of cetirizine and montelukast compared to the control group (p < 0.025). The study shows, for the first time, the steroid sparing effect of cetirizine and montelukast in addition to conventional treatment in MCNS patients with concomitant allergies.

## Introduction

Minimal-change nephrotic syndrome (MCNS) or minimal-change disease is one of the major causes of nephrotic syndromes. MCNS patients are generally responsive to corticosteroids and some may stay in remission after tapering off corticosteroids; however, roughly more than half of the patients experience relapse during their clinical course. These patients experience a variety of corticosteroid side effects.

The pathogenesis of MCNS remains obscure, but many clinical studies support T-cell abnormality in MCNS, which was initially proposed by Shalhoub in 1974. Recent studies report type 1 and 2 helper T-cell (Th1 and Th2, respectively) and type 17 helper T-cell and regulatory T-cell (Th17 and Treg, respectively) dysregulations in human MCNS. Th2 cytokine upregulation and Th1 cytokine downregulation in human MCNS patients has been suggested^1–6^, and Th17 cytokine upregulation and Treg downregulation has also been indicated^7–9^. Notably, the transcription level of GATA-binding protein 3 (GATA-3), a master regulator of Th2 differentiation and transcriptional activator of Th2 cytokines, such as interleukins (IL) 4, 5, and 13^10,11^, was significantly upregulated in peripheral blood mononuclear cells (PBMCs) from MCNS patients in the nephrotic state compared with remission state^1^. Meanwhile, retinoic acid receptor–related orphan receptor γt, a master regulator of Th17 cells, was upregulated and a major transcription factor in Treg cells, forkhead box P3 (Foxp3), was downregulated in PBMCs from MCNS patients^7^.

Likewise, Th1/Th2 and Th17/Treg imbalance with Th2 and Th17 predominance was reported in allergic diseases such as asthma^12,13^, atopic dermatitis^14^, and allergic rhinitis^15^. In allergic diseases, many cytokines and molecules, including histamine and leukotriene, have been investigated rigorously. Histamine plays an important role in building the Th2 state^16^ through both histamine H1^17^ and H2 receptors^18^ and the Th17 state through histamine H4 receptor^19^. Leukotriene also plays an important role in the allergic state shaping Th2^20^ and Th17^21^ predominance.

However, the therapeutic effect of anti-allergy treatment for MCNS has not been explored before. There is classical clinical knowledge that anti-histamines are frequently used for allergic rhinitis, anti-leukotrienes were also much used for asthma, and both drugs were used for urticaria. Scientifically, recent systematic review and meta-analysis have shown the following. Cetirizine is significantly effective for allergic rhinitis superior to montelukast^22,23^ whereas montelukast provides distinct benefit for asthma^24^. Both cetirizine^25^ and montelukast^26^ was effective for urticaria. For atopic dermatitis, which is thought to be strongly related to MCNS, beneficial evidence was observed for cyclosporin A^27^, which is a common immunotherapy also for MCNS. Therefore, in this observational study, we retrospectively investigated if anti-allergy treatment by histamine H1 receptor antagonist, cetirizine, combined with cysteinyl-leukotriene receptor antagonist, montelukast, is effective in disease control for adults with biopsy-proven MCNS.

## Methods

### Cohort definition

**Figure 1:**
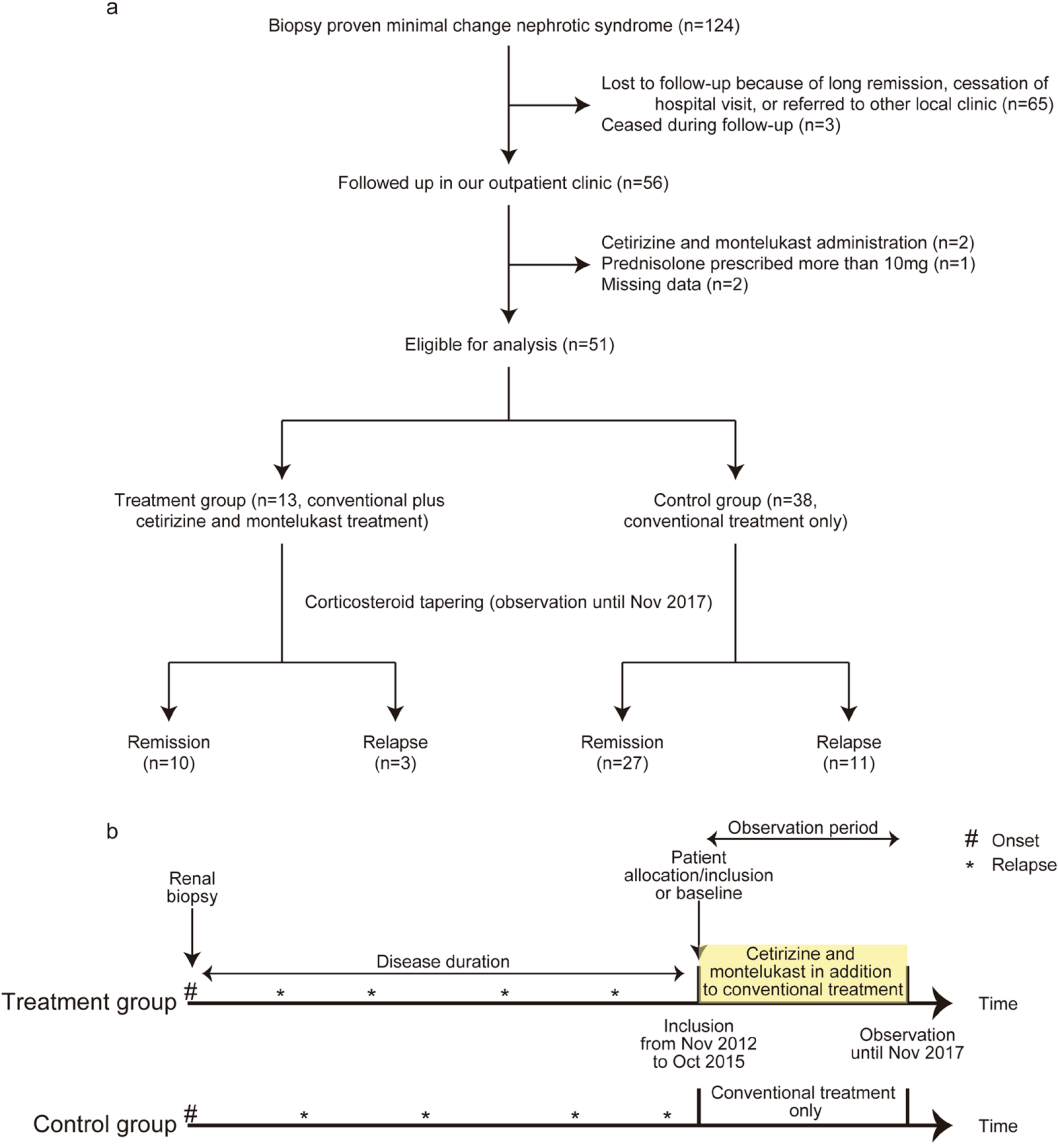
(**a**) Algorithm of this study. After excluding patients who were not eligible for analysis, 51 patients were included in the study. Thirteen patients who had been prescribed with cetirizine and montelukast were allocated to the treatment group, whereas the other 38 patients were allocated to the control group. After observation until November 2017, 10 (77%) patients in the treatment group remained in remission, compared with 27 (71%) patients in the control group. (**b**) Schematic diagram of this study for the two groups by timeline. Patients were included in the study as soon as they fulfilled the inclusion criteria between November 2012 and October 2015. Patients were followed up until November 2017.

Among 124 consecutive patients who presented with nephrotic syndrome and were diagnosed with new-onset or recurrent MCNS by renal biopsy from 1985 to 2015 at Toranomon Hospital, 65 patients were lost to follow-up by November 2017 (i.e., the end of follow-up) due to sustained complete remission of MCNS, cessation of hospital visit, or referral to other local clinics, and three patients died during the follow-up, resulting in 56 patients who were followed up until November 2017 in our outpatient clinic. We considered patients eligible for analysis if they met both of the following criteria between November 2012 and October 2015: (1) *de novo* exposure to cetirizine and montelukast, defined as initiation of these drugs in patients who had not been exposed to the drugs after the date of MCNS diagnosis and (2) disease remission and tapering of oral prednisolone to ≤ 10 mg/day. Therefore, 51 patients were included in the final cohort (Fig. 1a). Patients were further divided into two groups based on their exposure to cetirizine and montelukast: those treated with (treatment group, n = 13) and without (control group, n = 38) cetirizine and montelukast (Fig. 1b). Observation period is defined as the time of allocation/inclusion between November 2012 and October 2015 until November 2017 (i.e., the end of observation) (Fig. 1b). No *de novo* exposure was recorded between October 2015 and November 2017. The Toranomon Hospital Ethical Committee reviewed and approved the protocol (approval code 1739-H/B), and informed consent was obtained in the form of opt-out on the website. Those who rejected were excluded. All methods were performed in accordance with the Declaration of Helsinki.

### Definition of the outcomes

The minimum prednisolone dose was defined as the lowest daily dose of oral prednisolone throughout the treatment course, as shown in Fig. S1. Figure S1 shows tapering of prescribed prednisolone dose per day (vertical axis) during the time course (horizontal axis) in an example of an MCNS patient. First relapse as shown as an arrow with “Relapse 1” occurred when the prednisolone dose was 4 mg/day. Second relapse, “Relapse 2,” and third relapse, “Relapse 3,” occurred when the dose was 7.5 mg/day and 6 mg/day, respectively. Thus, the lowest daily dose of oral prednisolone throughout the treatment course is defined as 4 mg/day in this patient. Fig. S2a illustrates an example of successful reduction of the minimum prednisolone dose after the addition of cetirizine and montelukast treatment, and Fig. S2b shows an example of unsuccessful reduction of minimum prednisolone dose. In Fig. S2a, the patient was given cetirizine and montelukast added to conventional treatment in the yellow shaded period, resulting in successful tapering of prednisolone to corticosteroid free state, which leads the lowest daily dose of oral prednisolone throughout the treatment course to zero from 4 mg/day. In Fig. S2b, during the cetirizine and montelukast addition period shaded in yellow, fourth relapse, “Relapse 4,” occurred when prescribed prednisolone dose was 5 mg/day, which did not lower the minimum dose of 4 mg/day in the past. We scored this minimum dose both at baseline (before the yellow shaded period) and after the study (the whole period) for all patients and considered the treatment effective if the minimum dose is tapered after the study period. Co-primary outcomes were defined as (1) the lowest daily dose of oral prednisolone throughout the entire treatment course after the study compared to that of baseline, and (2) percentage of patients establishing corticosteroid-free state for the first time throughout the entire treatment course compared to the control group.

Other outcomes were defined as secondary outcomes as follows and used in sensitivity analysis. Relapse-free rate was depicted in Kaplan-Meyer curve for the respective groups and compared by log-rank analysis. Prednisolone dose on relapse was defined as prescribed prednisolone dose (mg/day) on relapse during the observation period (i.e. shaded period in yellow) in respective groups. Prednisolone tapering speed (mg/day/month) was defined as reduced prednisolone dose from baseline divided by the observed period until the first relapse during the follow-up or until November 2017.

We explored possible risk factors for relapse during the study period by single-factor Cox regression analysis and logistic regression analysis using the data from the control group before matching. The factors analyzed were age, estimated glomerular filtration rate (eGFR), urinary protein, number of past relapses per year, disease duration, other immunosuppressants, concomitant allergic disorders, minimum prednisolone dose, prednisolone dose at baseline, concomitant hypertension, dyslipidemia, diabetes mellitus, and hyperuricemia that required medication, serum IgE level, eosinophil count, and possible factors that aggravate MCNS such as infection (moderate or severe), irregular corticosteroid reduction, or new onset malignancy.

We also explored factors which have possible association with allergy complication for all patients at baseline by multivariate logistic regression analysis.

Supplementary methods describe clinical and histological diagnosis, treatment course, data collection, and statistical analyses.

## Results

### Propensity score matching

There was an imbalance of clinical characteristics between the two groups at baseline, as shown in Table S1. For comparison, we applied the 1:1 propensity score–matching method. We chose the following 10 factors for matching based on potential risk by clinical experience that might influence relapses and named this analysis as model 1: age, sex, eGFR, urinary protein, number of past relapses per year, follow-up length, other immunosuppressants, concomitant allergic disorders, minimum prednisolone dose, and prednisolone dose at baseline. Table 1 shows the values of each factor before and after propensity score matching, validated by standardized differences. It has been reported that a moderate standardized difference as large as 0.3 could still be consistent with the propensity score model having been correctly specified when the matched sample size is small^28^, as is applicable here. After matching with the above 10 factors, standardized differences improved enough so that the two groups were fair to compare. Only follow-up length could not be matched enough due to distinct differences between the two groups. The standardized difference for dyslipidemia, serum IgE level and possible MCNS aggravating factors (infection, irregular corticosteroid reduction, or new onset malignancy) were also improved after matching. More than 11 factors made the standardized difference too large, which did not make the model fair. The outcome was analyzed between the two groups after matching, as will be described in the following. Moreover, we selected two factors, serum IgE and possible MCNS aggravating factors (infection, irregular corticosteroid reduction, or new onset malignancy) which could influence MCNS disease stability and analyzed as propensity score matched model 2. The two groups were satisfactorily matched in terms of the two factors, although other factors were not matched enough as shown in Table S2.

**Table 1:** Clinical parameters before and after propensity score matching (model 1).

	Before matching			After matching (model 1)		
	Treatment (n = 13)	Control (n = 38)	Stand diff	Treatment (n = 13)	Control (n = 13)	Stand diff
*Age at inclusion, years	48.2 ± 14.0	54.8 ± 17.3	−0.42	48.2 ± 14.0	51.3 ± 17.2	−0.20
*Sex prevalence, no. of men %	7, 54%	25, 68%	−0.28	7, 54%	9, 69%	0.32
*eGFR at inclusion, ml/min/1.73 m^2^	86 ± 23	76 ± 17	0.49	86 ± 23	85 ± 11	0.09
*Urinary protein at inclusion, g/gCre	0.07 ± 0.12	0.12 ± 0.35	−0.19	0.07 ± 0.12	0.05 ± 0.07	0.20
Age at onset, years	31.1 ± 19.0	38.7 ± 19.0	−0.40	31.1 ± 19.0	35.0 ± 20.0	−0.20
Disease duration, months	205 ± 114	192 ± 156	0.09	205 ± 114	197 ± 112	0.09
Past relaplse, times	4.2 ± 3.3	2.7 ± 4.1	0.38	4.2 ± 3.3	4.3 ± 5.0	−0.04
*past relapse per year, times/yr	0.29 ± 0.19	0.15 ± 0.19	0.72	0.29 ± 0.19	0.27 ± 0.25	0.10
*Follow up period, months	44.6 ± 4.8	56.6 ± 10.2	−1.50	44.6 ± 4.8	54.7 ± 12.4	−1.07
*Other immunosupressants, no./%	6, 46%	10, 27%	0.41	6, 46%	7, 54%	−0.15
*Comcomitant allergic disorders, no./%	13, 100%	20, 54%	1.30	13, 100%	13, 100%	0.00
Dyslipedemia, no./%	1, 8%	14, 38%	−0.77	1, 8%	2, 15%	−0.24
*minimum PSL dose, mg	0.83 ± 1.1	2.6 ± 2.9	−0.37	0.83 ± 1.1	1.08 ± 2.7	−0.12
*PSL dose at baseline, mg	4.1 ± 3.7	3.3 ± 3.7	0.21	4.1 ± 3.7	4.1 ± 3.8	0.00
IgE, U/mL	780 ± 643	496 ± 592	0.46	780 ± 643	865 ± 676	−0.13
Eosinophil,/microL	80 ± 62	224 ± 198	−0.98	80 ± 62	258 ± 200	−1.20
Infection, irregular PSL reduction, or malignancy, no./%	4, 31%	16, 42%	−0.26	4, 31%	3, 23%	0.17

Values are presented as median ± standard deviation.

Stand diff, standardized difference; eGFR, estimated glomerular filtration rate; PSL, prednisolone; Cre, creatinine; *indicates items that were matched using the propensity-score analysis. *matched items.

### Results of the outcomes

**Figure 2:**
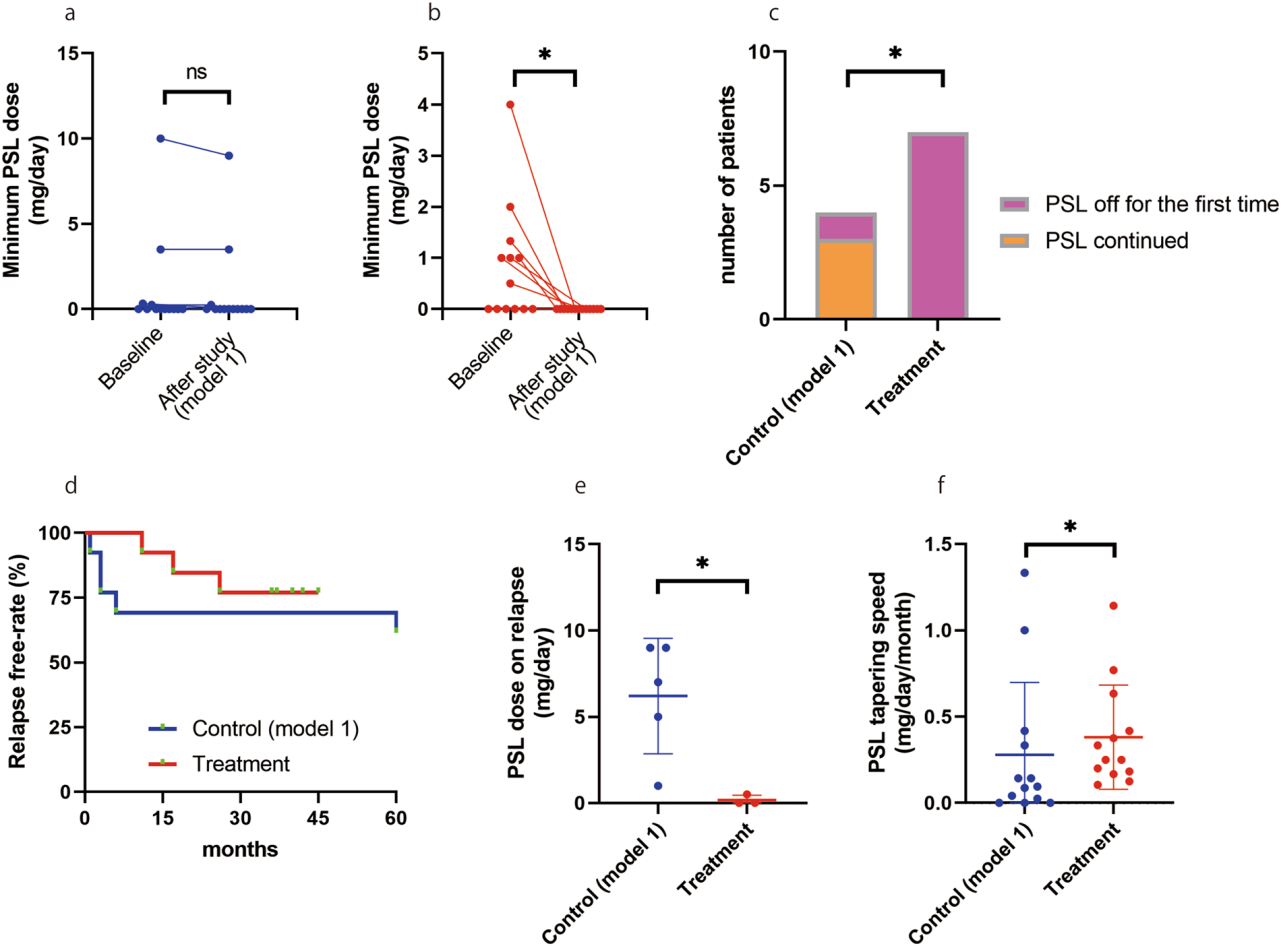
Results of the two groups after propensity score matching (model 1). (**a**,**b**) Lowest daily dose of oral prednisolone throughout the entire disease history for the respective groups. (**a**) No improvement on lowest prednisolone dose in disease history was observed in the control group (p = 0.37). (**b**) The improvement was confirmed by addition of cetirizine and montelukast on top of standard corticosteroid therapy (p = 0.0215). (**c**) The number of patients who reached PSL free state for the first time throughout their disease history. The fraction of patients who reached PSL free was significantly higher in the treatment group compared to the control group (p = 0.0242). (**d**) Survival curve of the two groups after propensity score matching. There was no statistical significance between the two groups by log-rank test (p = 0.53). (**e**) The prednisolone dose on relapse was significantly lower in the treatment group after propensity score matching (p = 0.035). (**f**) The prednisolone tapering rate was significantly higher by addition of cetirizine and montelukast (p = 0.048). *, p < 0.05; NS, not significant.

For the co-primary outcome (1), minimum prednisolone dose (i.e., the lowest daily dose of oral prednisolone throughout the disease course) was significantly reduced after the study period compared to that of the baseline in the treatment group (Fig. 2b, p = 0.0215), which was not observed in the control group (Fig. 2a, p = 0.37).

Table 2 shows the key clinical parameters of the propensity score–matched patients for the two groups (model 1). In the treatment group, excluding three patients who experienced relapse, 10 (77%) of 13 patients were maintained in corticosteroid-free remission for an average of 34 months at the end of the observation. The three patients who relapsed were administered rituximab for treatment. On the other hand, for the control group (model 1), 6 (46%) of 13 patients remained in corticosteroid-free remission because five patients experienced relapse and two patients were still on corticosteroids. Although there were almost twice as many patients who reached the corticosteroid-free remission in the treatment group compared to the control group, there was no statistically significant difference in overall percentage of patients between the two groups (77% vs 46%, p = 0.23). Notably, for the co-primary outcome (2), all seven who had never reached the corticosteroid-free remission (i.e., those whose minimum prednisolone dose was not zero) in the treatment group had established corticosteroid-free remission for the first time throughout the treatment course compared to one of four patients in the control group, which was statistically significant (Fig. 2c, 100% vs 25%, p = 0.0242). Among the treatment group, 1 (14%) of 7 patients who had never reached corticosteroid-free remission experienced relapse after prednisolone had been tapered off, whereas the remaining 6 (86%) patients were still in corticosteroid-free remission for an average of 34 months. Possible MCNS aggravating factors seen were herpes zoster virus reactivation, pyelonephritis, bronchiolitis, influenza, bladder cancer, rectum adenocarcinoma, and cervical cancer. Chronic side effects of corticosteroids seen in these patients through the entire disease course were cataracts, glaucoma, osteoporosis, osteonecrosis of the femoral head, alopecia, muscle weakness, infection, hypertension and obesity. Thus, both co-primary outcomes were statistically satisfied suggesting that cetirizine and montelukast combination has a steroid sparing effect.

**Table 2:** Characteristics of propensity score matched patients (model 1).

Group	Case	Age at baseline	Sex	Complicated allergy	Immuno-suppressants	PSL dose		Minimum PSL dose		Corticosteroid-free remission, months	IgE, U/mL	Infection, irregular corticosteroid reduction, or malignancy
						Baseline, mg	After the study, mg (PSL dose on relapse, mg)	Baseline, mg	After the study, mg			
Treatment (model 1)	1	62	F	AR	MZR	1	0	1	0	42	620	
	2	41	M	AD, As		1	relapse (0)	1	0	relapse (7)	12	
	3	45	M	AD, D	CyA	10	relapse (0.5)	0	0	relapse	1438	Bladder cancer, bronchiolitis
	4	30	M	AR, F	CyA	2	0	0	0	33	746	
	5	51	F	AD, S		0.5	0	0	0	37	182	
	6	38	M	AR		2	0	1	0	29	721	
	7	44	M	AD	MZR, CyA	1	0	0.5	0	35		
	8	39	F	AD, AR, D, F	Tac	10	relapse (0)	0	0	relapse (13)	33	Herpes zoster virus, influenza
	9	54	M	AR, S		10	0	0	0	25		
	10	32	F	AD, AR, S		3	0	2	0	37	1366	
	11	82	F	AD, D		8	0	4	0	37	93	Herpes zoster virus
	12	43	M	AD, AR		1.66	0	1.33	0	24	1937	
	13	65	F	AR	CyA	3	0	0	0	41	1430	Rectum adenocarcinoma
Control (model 1)	14	57	M	D	CyA	2	0	0	0	43		
	15	63	M	AR, U		4	0	0	0	14		
	16	40	F	D, F	CyA	8	relapse (7)	3.5	3.5	relapse		Cervical cancer
	17	76	M	As	CyA	0	0	0	0	60	1190	pyelonephritis
	18	64	F	AR		0.5	0	0.33	0	41	770	
	19	47	F	AD		0	0	0	0	60	1050	
	20	41	M	As	CyA	1.5	relapse (1)	0.25	0.25	relapse	2186	Rectum adenocarcinoma
	21	85	M	S		1.5	1.5	0	0	on PSL	181	
	22	34	M	As	CyA	10	relapse (9)	0	0	relapse		
	23	19	M	AR		10	relapse (5)	0	0	relapse	1650	
	24	42	M	D	CyA	10	relapse (9)	10	9	relapse	345	
	25	43	M	U		3	2	0	0	on PSL	195	
	26	56	F	AR, D	CyA	2.5	0	0	0	54	220	

Clinical characteristics of propensity score–matched patients (model 1).

AD, atopic dermatitis; AR, allergic rhinitis; As, asthma; S, sinusitis; D, drug allergy; F, food allergy; U, urticaria; PSL, prednisolone; CyA, cyclosporin A; MZR, mizoribine; Tac, tacrolimus.

Secondary outcomes as described as follows were used as sensitivity analyses. Figure 2d shows that there was no significant difference in relapse between the two groups after propensity score matching, as evaluated by log-rank analysis (p = 0.53). As shown in Fig. 2e, however, the treatment group had significantly lower prednisolone dose on relapse compared with that of the control group (p = 0.036), supporting that cetirizine and montelukast possess a steroid sparing effect. This was also consistent with the result that the prednisolone tapering speed was significantly higher in the treatment group compared to the control group (Fig. 2f, p = 0.048), because fast tapering of corticosteroids triggers relapse in general clinical settings. As for histological features between with and without allergies, there were no apparent difference that were detectable. All the kidney histology looks the same with no apparent changes in glomeruli and interstitium. Interstitial fibrosis looks to be dependent on underlying conditions such as benign nephrosclerosis and aging that are independent of MCNS. Only diffuse podocyte foot process effacement is seen in electron microscopy. In fact, in the matched model 1, eGFR levels which is closely associated with interstitial fibrosis are almost the same between the two groups as shown in Table 1.

We analyzed the single-factor Cox regression analysis for relapse in the overall control group before matching to detect the possible factors associated with relapse during conventional treatment only in our MCNS population. The higher number of past relapses per year, immunosuppressant administration, and younger age at baseline were significantly associated with relapse, as shown in Table 3, implying that these three factors may be associated with high disease activity during the conventional treatment course. Logistic regression analysis of relapse showed similar results as shown in Table S4. Immunosuppressants were given for frequently relapsing patients in our hospital based on decisions of clinical experts. Because only 11 patients among the 38 patients in the overall control group experienced relapse, we could not apply multivariable analysis due to a lack in the number of events. The result supports that the selection of the matching items were valid in our population because all the three factors were included in the 10 items used for propensity score matching model 1. The treatment group was excluded because anti-allergy agents may influence relapse in this case.

**Table 3:** Coefficient of Univariate analysis by cox regression analysis of relapse during observation for patients without anti-allergy therapy.

Variable	Hazard ratio	Lower 95% CI	Upper 95% CI	p value
Past relapse per year, 1 time per year	37.6	2.5	568.5	0.009
Immunosupressant, yes	4.0	1.2	13.2	0.022
Age, 10 years	0.67	0.47	0.97	0.032
Disease duration, 1 year	0.97	0.92	1.02	0.26
Urinary protein, 1 g/gCre	0.28	0.00	37.0	0.61
eGFR, 1 ml/min/1.73 m2	1.01	0.97	1.04	0.81
Minimum PSL dose, 1 mg	1.03	0.84	1.26	0.78
Concomitant allergic disorders, yes	1.08	0.33	3.57	0.89
PSL dose at baseline, 1 mg	1.11	0.95	1.29	0.18
Dyslipedemia, yes	0.54	0.14	2.03	0.36
Hyperurecemia, yes	0.75	0.10	5.87	0.78
Hypertension, yes	1.04	0.32	3.42	0.94
Diabetes mellitus, yes	1.77	0.54	5.81	0.35
Infection, irregular corticosteroid reduction, or malignancy, yes	1.40	0.30	6.46	0.67
IgE, 1 U/mL	1.00	1.00	1.00	0.40
Eos, 1/microg	1.00	1.00	1.00	0.52

Coefficients of univariate Cox regression analysis of relapse during observation in the control group.

Past relapse per year, use of immunosuppressants, and age were statistically associated with relapse.

Cre, creatinine; eGFR, estimated glomerular filtration rate; PSL, prednisolone; CI, confidence interval.

The results for matching model 2 are shown in Table S3 and Fig. S3. Minimum prednisolone dose was reduced (Fig. S3b, p = 0.034) and more patients had achieved prednisolone free state for the first time (Fig. S3c, p = 0.046) compared to the control group, which support the results of matching model 1. Although, the statistically significant level of p < 0.025 was not satisfied for these co-primary outcomes.

Table S5 shows the patient characteristics by separating if they have concomitant allergies or not at baseline. Patients with concomitant allergies had characteristics as follows: significantly higher eGFR, younger age, higher past relapse per year, higher percentage of cyclosporin A use, and less treatment-required dyslipidemia. We conducted univariate logistic regression analysis for concomitant allergy and found that patients with allergy had higher past relapse per year, more treatment-required hypertension, and tended to have less treatment-required dyslipidemia, as shown in Table S6. Multivariate logistic regression analysis showed that the higher past relapse per year and more frequent treatment-required hypertension was associated with allergy presence in MCNS patients as shown in Table 4.

**Table 4:** Coefficient of multivariate analysis by logistic regression analysis of concomitant allergy disorders for all patients.

Variable	Odds ratio	Lower 95% CI	Upper 95% CI	p value
Past relapse per year, 1 time per year	720	1.58	329000	0.035
Hypertension, yes	10.9	1.41	85	0.022
Dyslipedemia, yes	0.14	0.018	1.07	0.058

Coefficient of multivariate logistic regression analysis of concomitant allergy disorders for all patients.

Past relapse per year and presence of treatment-required hypertension were statistically associated with concomitant allergy in MCNS patients.

## Discussion

We focused on the frequent complications of allergic diseases in MCNS and analyzed if anti-allergy treatment could help control the disease activity of MCNS. We have selected patients by highly specific criteria composed of clinical diagnosis of nephrotic syndrome and pathological diagnosis of MCNS, so that the effect of additional therapy by cetirizine and montelukast could be assessed effectively. Through propensity score matching, we show, for the first time, that the two agents significantly reduced the required corticosteroid dosage for maintaining remission in repeated relapsing MCNS patients with concomitant allergic disorders, thus showing steroid sparing effect.

The effect of cetirizine or montelukast on corticosteroid dependency or relapse in MCNS patients have not been described thus far. Zedan *et al*.^29^ conducted a randomized control study in childhood-onset nephrotic syndrome patients to determine if montelukast has beneficial effects on idiopathic nephrotic syndrome, but they did not evaluate renal biopsies, corticosteroid dosage, and relapse profiles. Instead, they showed that plasma leukotriene B4 and LTC4/D4/E4 were significantly decreased by montelukast treatment^29^.

Here in the propensity score-matching model 1 as shown in Fig. 2, both the co-primary outcomes was statistically achieved: reduction in the lowest daily dose of oral prednisolone throughout the entire treatment course after the study compared to that of baseline (p < 0.025), and higher percentage of patients establishing corticosteroid-free state for the first time throughout the entire treatment course compared to the control group (p < 0.025). The idea of steroid sparing effect of cetirizine and montelukast was also supported by significant difference in prednisolone dose on relapse (Fig. 2e) and corticosteroid tapering speed (Fig. 2f). As shown in Table 1, the clinical characteristics of the two groups were satisfactorily similar at baseline. Other factors that were not included in the ten factors used in model 1 such as IgE or possible MCNS aggravating factors (infection, irregular corticosteroid reduction, or new onset malignancy) was also similar at baseline, although some of the IgE data were missing. Eosinophil data were missing for 41 patients, so we could not apply for propensity score-matching analysis. To focus on serum IgE level and possible MCNS aggravating factors that may influence MCNS relapse as reported previously^30–34^, we created matching model 2 which results are shown in Table S2. Mostly different patients were selected for model 2 in the control group, and only four patients were common in both model 1 and model 2 control groups. Patients with missing IgE were automatically excluded. If the two factors in model 2 had significant impact on relapse or corticosteroid sparing effect which overwhelms the therapeutic effect of cetirizine and montelukast, no therapeutic trend would be observed after the treatment. Contrary to this as shown in Fig. S3, results of the model 2 were similar to that of model 1 which supported the results obtained in model 1. Although not statistically significant, trends were seen for the following. Minimum prednisolone dose was reduced after the observation, percentage of patients whose prednisolone was off for the first time was higher, prednisolone dose on relapse was lower, and prednisolone tapering rate was higher in the treatment group compared to the control group, respectively. Altogether, in this propensity score-matching model as shown by model 1 and supported by model 2, cetirizine and montelukast have suggested for the first time the corticosteroid sparing effect in allergy concomitant MCNS patients.

In clinical settings, we occasionally encountered seasonal recurrence of MCNS occurring simultaneously with seasonal rhinitis caused by pollens from cryptomeria or cypress; this finding is consistent with a previous report^35^. Some of our patients reported fewer complaints, and some even experienced relief from symptoms, rhinitis and/or itchiness from atopic dermatitis by the two medications. These symptomatic improvements may be associated with better MCNS disease control for allergy-complicated MCNS patients. Regarding patient safety, no serious or minor side effects were noted.

Additionally, we searched for possible factors associated with relapse and allergy, respectively. For relapse, we applied univariate logistic regression or cox regression analysis for patients without anti-allergy therapy, and the results are shown in Tables S4 and 3, respectively. Both analyses returned similar results. Possible association was observed for past relapse per year, immunosuppressant use, and age all of which was included in the ten factors for matching in model 1. We could not obtain a definite formula for relapse because multivariate analysis could not be applied due to small number of relapses. For factors associated with allergy, results of univariate and multivariate logistic regression analysis are shown in Tables S6 and 4, respectively. These results indicate that frequent relapse may be associated with concomitant allergies, thus suggesting that anti-allergy therapy may have possible benefit for controlling MCNS disease activity. Treatment-required hypertension was also associated with allergies, although whether this association involves hypertension itself or anti-hypertensive drugs is unknown. Regardless of the fact that IgE level is increased in patients with allergy disorders in general, it is interesting to see that IgE level was not associated with allergy in this analysis. This suggested that MCNS itself may have increased IgE level by its intrinsic pathophysiology. Possible molecular mechanisms of leukotriene, histamine, and MCNS pathophysiology are discussed in the supplementary material.

This study had following limitations. First, the patient characteristics that were included indicate that the treatment was effective for a confined population of multiple-relapse and allergy-concomitant MCNS patients with prolonged disease duration. It is important that many patients who reached remission without relapse or who had reached long remission were not included (Fig. 1a). The average disease duration was as long as 200 months in the respective groups (Table 1). Second, the effect of older age may have influenced the reduced required corticosteroid dose because younger age had been identified as a possible risk factor in the single-factor Cox regression model for the conventional treatment population (Table 3). However, the reduction of the lowest daily dose of oral prednisolone throughout disease course, i.e. minimum prednisolone dose, was significantly reduced only in the treatment group (Fig. 2a,b), suggesting that there was superiority in adding cetirizine and montelukast for disease control compared to conventional treatment only. Third, the benefits gained by immunosuppressants were not completely adjusted because we could only consider if patients took immunosuppressants or not. The difference in effectiveness among the three types of immunosuppressants (cyclosporin A, mizoribine, or tacrolimus) for MCNS has not been reported, as far as we know. Fourth, the follow-up period was shorter in the treatment group compared to the control group. Although this may lead to increased relapse rate or lower prednisolone tapering speed in the treatment group to some extent; this effect would be limited because the covered follow-up period by the treatment group was as large as 82% of the period achieved by the control group. Moreover, a shorter follow-up period would not influence the co-primary outcomes, i.e. minimum prednisolone dose after follow-up or percentage of corticosteroid free condition establishment for the first time throughout the entire disease in an unfavorable manner in the presented design. Fifth, regarding on regression analysis for relapse, we could not apply multivariate analysis because there were not enough relapse cases. Lastly, this study was not a randomized control trial, and its small sample size was also a shortcoming, therefore the accuracy of the conclusion may be affected. On the other hand, the strengths of presented data could be stated as follows. First, the biopsy findings and clinical diagnosis of nephrotic syndrome supports the definite diagnosis of MCNS and excludes apparent FSGS. Second, data of long-term follow up is scares in general, therefore average follow up of 200 months at inclusion and average follow up of 50 months for observation is valuable. These two features are expected to contribute to the improved accuracy of the collected data. Also, we have conducted additional propensity score matching (model 2) analysis to validate for other possible confounding factors associated with MCNS relapse which are serum IgE and “possible MCNS aggravating factors (infection, irregular glucocorticoid reduction, and new onset malignancy)”. Model 2 showed similar results (Fig. S3) providing a supportive data for model 1.

In conclusion, the addition of cetirizine and montelukast treatment for MCNS patients with prolonged disease duration concomitant with allergic disorders was effective in reducing daily corticosteroid dosage. The effects of the two agents on MCNS should be validated in other populations. Furthermore, whether these medications are effective for early-phase MCNS patients, for patients without concomitant allergies, or for MCNS patients in general, should be elucidated in future studies.

## Supplementary information

Supplementary information.

## Data Availability

No restrictions exist regarding the availability of data. Readers can obtain materials and information through contacting corresponding author.
