# Supplementary material for: Corticosteroid reduction by addition of cetirizine and montelukast in biopsy-proven minimal-change nephrotic syndrome concomitant with allergic disorders
Source: Sci Rep. 2020 Jan 30;10:1490. doi: 10.1038/s41598-020-58463-z (PMC6992583; doi:10.1038/s41598-020-58463-z)
Supplement: Supplementary file 1 — Supplementary information. [file 41598_2020_58463_MOESM1_ESM.pdf]

Supplementary Material

Corticosteroid reduction by addition of cetirizine and montelukast in biopsy-proven minimal-change nephrotic syndrome concomitant with allergic disorders

Yoichi Oshima[1,2], Keiichi Sumida[1,3], Masayuki Yamanouchi[1], Noriko Hayami[1], Akinari Sekine[1], Hiroki Mizuno[1], Masahiro Kawada[1], Rikako Hiramatsu[1], Eiko Hasegawa[1], Tatsuya Suwabe[1], Junichi Hoshino[1], Naoki Sawa[1], Takeshi Fujii[4], Kenmei Takaichi[1,5], Yoshifumi Ubara[1,5]

[1]Nephrology Center, Toranomon Hospital, Tokyo, Japan

[2]Department of Nephrology, Endocrinology, and Metabolism, Keio University School of Medicine, Tokyo, Japan

[3]Division of Nephrology, Department of Medicine, University of Tennessee Health Science Center, Memphis, TN, USA

[4]Department of Pathology, Toranomon Hospital, Tokyo, Japan

[5]Okinaka Memorial Institute for Medical Research, Toranomon Hospital, Tokyo, Japan

Running title: Anti-allergy therapy in MCNS

Corresponding author: Yoichi Oshima, MD

Nephrology Center, Toranomon Hospital, 2-2-2, Toranomon, Minato, Tokyo 105-8470, Japan

E-mail: yoichi-o-s@hotmail.co.jp

Key words: Minimal-change nephrotic syndrome, allergy, montelukast, cetirizine, corticosteroid

Supplementary Methods

Clinical and Histological Diagnosis

Nephrotic syndrome was diagnosed based on the following criteria at disease onset: serum total protein <6.0 g/dL; serum albumin <3.0 g/dL; proteinuria >3.5 g/24 hour collected urine (g/day) or 3.5 g/g creatinine (Cre) by spot-collected urine; edema on the face, eyelid, neck, trunk, arms, or legs. Patients lacking any of the above four criteria were excluded from the study even if they satisfied the histological characteristics of MCNS.

The renal biopsy specimens were histologically analyzed by professional pathologists. Histological MCNS was diagnosed based on the following: no glomerular abnormalities in the mesangium, glomerular basement membrane, capillaries, podocytes, and Bowman's capsules through hematoxylin and eosin staining, periodic acid–Schiff, and periodic acid–methenamine silver staining under light microscope; no glomerular positive staining of IgG, IgA, IgM, C3, C4, or C1q by immunofluorescent staining (mild IgA or IgM staining in the mesangial area was allowed); no findings that support other causes of nephrotic syndrome such as focal segmental glomerulosclerosis, membranous glomerulopathy, amyloidosis, or membranous proliferative glomerulonephritis under light microscope. Additionally, we conducted electron microscopic analysis for cases that had to be differentiated from other electron deposition diseases. Cases analyzed by electron microscopy showed findings consistent with MCNS, i.e., diffuse podocyte foot process effacement.

Treatment course

To maintain disease control, oral corticosteroids, mainly prednisolone, were used, and very few patients received methylprednisolone or betamethasone. All patients were followed up in our outpatient clinic at 1- to 3-month intervals. In most patients, the daily dose of oral prednisolone was tapered by 2.5 to 5 mg at every visit until reaching ~20 mg/day and by 0.5 to 2.5 mg at every visit until reaching ~10 mg/day. Subsequently, prednisolone was tapered more slowly and cautiously to reach the lowest dose possible, depending on patient conditions. Some patients had been prescribed with immunosuppressants, such as cyclosporin A, bredinin, or tacrolimus, while using corticosteroids for further disease control based on the decision of their primary nephrologist. No immunosuppressants were added during the observation period.

Data collection

We collected the data by reviewing medical records retrospectively. Collected data included onset age, sex, status at time of presentation to our hospital (remission, relapse, or onset), laboratory data (serum total protein, albumin, urea nitrogen, Cre, IgG, IgA, IgM, IgE, eosinophils, urinary protein) on disease onset, disease duration at inclusion from onset, number of past relapses, follow-up period after inclusion, concomitant allergic disorders (rhinitis, atopic dermatitis, sinusitis, drug allergy, food allergy, asthma, urticaria), other immunosuppressants (cyclosporin A, bredinin, or tacrolimus), and concomitant hypertension, dyslipidemia, diabetes mellitus, and hyperuricemia that required

medication, infection, irregular corticosteroid reduction, or new onset malignancy. Relapse was considered as proteinuria level ≥1.0 g/g Cre (or g/day) that required an increased corticosteroid dose for disease control. Four milligrams of methylprednisolone and 0.8 mg of betamethasone were calculated to be equivalent to 5 mg of prednisolone according to a commonly used corticosteroid conversion rate.

Statistical analyses

Baseline patient characteristics were presented as number (percentage) for categorical variables and median (interquartile range) for continuous variables. Categorical and continuous variables were compared using Fisher's exact test and Mann-Whitney U test, respectively. Wilcoxon signed-rank test was used for comparison before and after treatment. Cox regression analysis and logistic regression analysis was used for possible risk factors of relapse. Univariate or multivariate losgistic regression analysis was used for factors associated with the presence of allergic disorders. Relapse free survival between patients with and without anti-allergic treatment was assessed with Kaplan-Meier and log-rank analyses. To account for the confoundment arising from different clinical characteristics of patients treated with (vs. without) cetirizine and montelukast, we performed a propensity score–matched analysis as our primary analysis. Propensity scores for the likelihood of presence versus absence of cetirizine and montelukast treatment were calculated by logistic regression using ten variables at baseline (i.e., the date of patient inclusion) ; age, sex, eGFR, urinary protein,

number of past relapses per year, follow-up length, other immunosuppressants, concomitant allergic disorders, minimum prednisolone dose, and prednisolone dose at baseline. We then matched patients with the anti-allergic treatment with comparable patients without the treatment using a 1:1 nearest-neighbor matching without replacement. Differences between variables were examined by calculating standardized differences, and values <0.3 were considered acceptable for the matching. We also conducted propensity score method using two variables, serum IgE and MCNS aggravating factors (infection, irregular corticosteroid reduction, or new onset malignancy), to focus on possible clinically relevant MCNS relapse. For co-primary outcomes, we splitted alpha into two in this study. The reported p values are two sided and reported as significant at <0.025 for the two primary outcomes. Other outcomes were considered significant at p value of <0.05. The patients were followed-up until November 2017. All statistical analyses were performed with EZR version 1.35 (Saitama Medical Center, Jichi Medical University, Saitama, Japan), which is a graphical user interface for R (The R Foundation for Statistical Computing, Vienna, Austria).[1]

Supplementary Results

Clinical characteristics

The characteristics of the two groups (control and treatment groups) are summarized in Table S1 upon allocation (i.e., the baseline). There were no differences in age, sex, and laboratory data including IgE and eosinophil levels at disease onset. There were no differences in eGFR, urinary protein, disease duration, age, or use of other immunosuppressants within the study duration between the two groups. The numbers of past relapses and past relapses per year were significantly higher in the treatment group. The follow-up period was significantly higher in the control group because patients in the treatment group tended to satisfy the inclusion criterion (the prescription of cetirizine and montelukast) later in the inclusion window period. The prevalence of allergic disorders was significantly higher in the treatment group. Among the allergic diseases, rhinitis, atopic dermatitis, and sinusitis were significantly more prevalent in the treatment group. For medication-requiring concomitant diseases, dyslipidemia was significantly more prevalent in the control group, but no difference was seen for hypertension, diabetes mellitus, or hyperuricemia. The minimum prednisolone dose or prescribed prednisolone dose at baseline was similar between the two groups.

Supplementary Description

Possible molecular mechanism of contribution of anti-allergy treatment in MCNS disease stability.

IL-13, categorized as a Th2 cytokine, is reported to have a role in MCNS pathophysiology. Xue et al.[2,3] showed that LTE4 induces IL-13 and other Th2 proinflammatory cytokines in human Th2 cells. Yap et al.[4] have reported that the IL-13 mRNA level is significantly upregulated in CD4-positive T cells from MCNS children in relapse state. Lai et al.[5] showed that IL-13 overexpression in rats provokes MCNS-like nephropathy with diffuse foot process effacement, hypoalbuminemia, and proteinuria. Chan et al.[6] have shown that in cultured human podocytes, IL-13 induces phosphorylation of Vav1, a guanine nucleotide exchange factor of the Rho family, and activated Rac1 activity, resulting in actin cytoskeleton rearrangements. They also showed that Vav1 in podocytes were phosphorylated in IL-13 transgenic rats, suggesting the activation of Vav1 in an IL-13 overexpression model. Recently, Robins et al.[7] have also shown that Rac1 overexpression in podocytes using a podocyte-specific Rac1 transgenic mouse model and doxycycline-inducible system resulted in nephrotic syndrome. They also showed that Rac-guanosine triphosphate (GTP) stained positive in podocytes in kidney biopsy specimens from human MCNS patients. All of these results suggest the possible pathogenesis of MCNS; Th2 predominance and IL-13 upregulation cause Vav1-Rac1 pathway activation, leading to podocyte cytoskeleton dysregulation and eventual MCNS. Additionally, Ha et al.[8] treated cultured human podocytes with IL-13 and showed loss of cell processes or projections as well as downregulation of slit diaphragm proteins CD2AP, synaptopodin, or ZO-1, which were significantly

reversed by montelukast treatment. This suggests that montelukast has a direct effect in restoring podocyte dysregulation by IL-13. Therefore, montelukast may have a dual role in protecting patients from MCNS by inhibiting T cells from producing Th2 cytokines such as IL-13 and protecting podocytes from dysregulation caused by IL-13.

No study thus far has reported on the effects of histamine H1 receptor blockade for MCNS patients. Although histamine plays a pivotal role in allergies and its action on T lymphocytes is not elucidated enough because of highly complicated regulations by different cell types,[9,10] its role on Th2 cytokine IL-13 has been described. Elliot et al.[11] showed that Th2 cells treated with histamine increased IL-13 production through H1 and H2 receptors. According to Okamoto et al.,[12] H1 receptor antagonist blocks Th2 cytokine production. H1 receptor antagonist cetirizine may have therapeutic effects in MCNS patients due to its immunomodulating properties, as stated above.

Supplementary Table Legends

Table S1. Clinical characteristics of the two groups before matching by propensity score analysis.

Values are presented as interquartile range, median [25th percentile-75th percentile] for continuous variables. a, n=24; b, n=16; c, n=5; d, n=23; e, n=37.

eGFR, estimated glomerular filtration rate; PSL, prednisolone; Cre, creatinine.

Table S2. Clinical parameters before and after propensity score matching (model 2).

Values are presented as median ± standard deviation.

Stand diff, standardized difference; *indicates items that were matched using the propensity-score analysis

Table S3. Characteristics of propensity score matched patients (model 2)

AD, atopic dermatitis; AR, allergic rhinitis; As, asthma; S, sinusitis; D, drug allergy; F, food allergy; U, urticaria; PSL, prednisolone; CyA, cyclosporin A; MZR, mizoribine; Tac, tacrolimus.

Table S4. Coefficient of univariate analysis by logistic regression analysis of relapse during observation for patients without anti-allergy therapy.

Table S5. Characteristics of patients with or without allergy disorders.

Table S6. Coefficient of univariate analysis by logistic regression analysis of concomitant allergy disorders for all patients.

Supplementary Figure Legends

Figure S1. Schematic diagram of minimum prednisolone (PSL) dose. The minimum PSL dose for this patient was 4 mg, which was the lowest daily dose of oral prednisolone throughout the treatment course.

Figure S2. Example of minimum prednisolone (PSL) dose after addition of cetirizine and montelukast. a. The blue circle shows the PSL dose at inclusion or baseline. The minimum PSL dose for this patient was lowered after the addition of the two drugs. b. The minimum PSL dose for this patient was not lowered.

Figure S3. Results of the two groups after propensity score matching (model 2). (d) Survival curve of the two groups after propensity score matching. There was no statistical significance between the two groups by log-rank test ($p=0.86$). (e) The prednisolone dose on relapse was not significantly lower in the treatment group after propensity score matching ($p=0.10$). (f) The prednisolone tapering rate was not significantly lower by addition of cetirizine and montelukast ($p=0.161$). (d, e) Lowest daily dose of oral prednisolone throughout the entire disease history for the respective groups. (d) No improvement on lowest prednisolone dose in disease history was observed in the control group ($p=0.174$). (e) The improvement was confirmed by addition of cetirizine and montelukast on top of standard corticosteroid therapy ($p=0.034$), although not satisfying $p<0.025$. (f) The number of patients who reached PSL free state for the first time throughout their disease history. The fraction of patients who reached PSL free was higher in the treatment group compared to the control group ($p=0.046$),

although not satisfying $p<0.025$.

Supplementary References

1 Kanda, Y. Investigation of the freely available easy-to-use software 'EZR' for medical statistics. *Bone Marrow Transplant.* **48**, 452-458, doi:10.1038/bmt.2012.244 (2013).

2 Xue, L. *et al.* Prostaglandin D2 and leukotriene E4 synergize to stimulate diverse TH2 functions and TH2 cell/neutrophil crosstalk. *J. Allergy Clin. Immunol.* **135**, 1358-1366.e1351-1311, doi:10.1016/j.jaci.2014.09.006 (2015).

3 Xue, L. *et al.* Leukotriene E4 activates human Th2 cells for exaggerated proinflammatory cytokine production in response to prostaglandin D2. *J. Immunol.* **188**, 694-702, doi:10.4049/jimmunol.1102474 (2012).

4 Yap, H. K. *et al.* Th1 and Th2 cytokine mRNA profiles in childhood nephrotic syndrome: evidence for increased IL-13 mRNA expression in relapse. *J Am Soc Nephrol* **10**, 529-537 (1999).

5 Lai, K. W. *et al.* Overexpression of interleukin-13 induces minimal-change-like nephropathy in rats. *J Am Soc Nephrol* **18**, 1476-1485, doi:10.1681/asn.2006070710 (2007).

6 Chan, C. Y. *et al.* Novel role of Vav1-Rac1 pathway in actin cytoskeleton regulation in Interleukin-13-induced minimal change-like nephropathy. *Clin. Sci. (Lond.)*, doi:10.1042/cs20160312 (2016).

7 Robins, R. *et al.* Rac1 activation in podocytes induces the spectrum of nephrotic syndrome. *Kidney Int* **92**, 349-364, doi:10.1016/j.kint.2017.03.010 (2017).

8 Ha, T. S. *et al.* Montelukast improves the changes of cytoskeletal and adaptor proteins of human podocytes by interleukin-13. *Inflamm. Res.* **66**, 793-802, doi:10.1007/s00011-017-1058-y (2017).

9 Thurmond, R. L., Gelfand, E. W. & Dunford, P. J. The role of histamine H1 and H4 receptors in allergic inflammation: the search for new antihistamines. *Nature reviews. Drug discovery* **7**, 41-53, doi:10.1038/nrd2465 (2008).

10 Kmiecik, T., Otocka-Kmiecik, A., Gorska-Ciebiada, M. & Ciebiada, M. T lymphocytes as a target of histamine action. *Arch. Med. Sci.* **8**, 154-161, doi:10.5114/aoms.2012.27295 (2012).

11 Elliott, K. A., Osna, N. A., Scofield, M. A. & Khan, M. M. Regulation of IL-13 production by histamine in cloned murine T helper type 2 cells. *Int Immunopharmacol* **1**, 1923-1937 (2001).

12 Okamoto, T., Iwata, S., Ohnuma, K., Dang, N. H. & Morimoto, C. Histamine H1-receptor antagonists with immunomodulating activities: potential use for modulating T helper type 1 (Th1)/Th2 cytokine imbalance and inflammatory responses in allergic diseases. *Clin Exp Immunol* **157**, 27-34, doi:10.1111/j.1365-2249.2009.03958.x (2009).

Table S1. Clinical characteristics of patients by groups

	treatment (n=13)	control (n=38)	p
At onset			
Age, years	24.0 [19.0-39.0]	39.0 [23.3-52.8]	0.15
Sex, male/female	7/6	25/13	0.52
Laboratory data (onset cases only)	n=6	n=25	
Total protein, g/dL	4.2 [3.7-4.6]	4.3 [4.0-4.9]	0.45
Albumin, g/dL	1.5 [1.1-2.0]	1.6 [1.1-1.8]	0.74
Urea nitrogen, mg/dL	20.5 [11.3-23.8]	16.0 [12.0-21.0]	0.84
Creatinine, mg/dL	0.7 [0.6-1.1]	0.9 [0.8-1.0]	0.30
IgG, mg/dL	523 [442-803]	481 [375-711], a	0.59
IgA, mg/dL	320 [298-403]	272 [179-369], a	0.19
IgM, mg/dL	153 [120-185]	144 [104-210], a	0.90
IgE, IU/mL	1044 [646-1414]	249 [115-707], b	0.13
Eosinophil, cells/mcL	116 [94-150], c	197 [119-315], d	0.07
Urinary protein, g/day	16.2 [9.2-17.5], c	8.4 [6.6-10.7]	0.42
At inclusion			
eGFR, ml/min/1.73m^2	83.0 [70.3-102.0]	74.5 [67.6-88.6], e	0.19
Urinary protein, g/gCre	0.03 [0.01-0.06]	0.03 [0.03-0.06], e	0.56
Disease duration, months	160 [139-248]	158 [80-289]	0.63
Age, years	44.0 [39.0-54.0]	56.5 [42.0-65.8]	0.23
Past relapse, times	3.0 [2.0-4.0]	1.0 [0-3.8]	0.016
Past relapse per year, times/yr	0.167 [0.180-0.389]	0.0 [0.079-0.260]	0.004
Follow up period, months	44 [41-46]	60.0 [60.0-60.0]	<0.001
Other immunosupressants, no./%	6, 46%	10, 26%	0.30
Cyclosporin, no./%	4, 31%	9, 24%	0.72

Bredinin, no./%	2, 15%	1, 3%	0.16
Tacrolimus, no./%	1, 8%	0, 0%	0.26
Comcomitant allergic disorders, no./%	13, 100%	20, 53%	0.002
Rhinitis, no./%	8, 62%	7, 18%	0.01
Atopic dermatitis , no./%	8, 62%	1, 3%	<0.001
Sinusitis, no./%	3, 23%	1, 3%	0.046
Drug allergy, no./%	3, 23%	7, 18%	0.70
Food allergy, no./%	2, 15%	0, 0%	0.06
Asthma, no./%	1, 8%	4, 11%	1.00
Urticaria, no./%	0, 0%	3, 8%	0.56
Medication required comcomitant disease, no./%	7, 54%	27, 71%	0.32
Hypertension, no./%	6, 46%	20, 53%	0.76
Dyslipidemia, no./%	1, 8%	15, 39%	0.04
Diabetes mellitus, no./%	1, 8%	12, 32%	0.14
Hyperurecemia, no./%	3, 23%	4, 11%	0.35
Least PSL dose in disease history, mg	0.5 [0-1.0]	0.0 [0.0-2.0]	0.96
PSL dose at inclusion, mg	2.0 [1.0-8.0]	2.0 [0.0-5.0]	0.28
MCNS aggravating factors (infection, irregular corticosteroid reduction, or malignancy), no./%	4, 31%	16, 42%	0.21
Infection, no./%	3, 23%	12, 32%	0.17
Irregular corticosteroid reduction, no./%	0, 0%	0, 0%	
Malignancy, no./%	2, 15%	4, 11%	0.28

Table S2

	Before matching			After matching (model 2)		
	treatment (n=13)	control (n=38)	Stand diff	treatment (n=11)	control (n=11)	Stand diff
Age at inclusion, years	48.2 ± 14.0	54.8 ± 17.3	-0.42	48.0 ± 15.1	49.7 ± 18.5	-0.10
Sex prevalence, no. of men %	7, 54%	25, 68%	-0.28	5, 45%	7, 64%	0.37
eGFR at inclusion, ml/min/1.73m2	86 ± 23	76 ± 17	0.49	84 ± 24	77 ± 11	0.41
Urinary protein at inclusion, g/gCre	0.07 ± 0.12	0.12 ± 0.35	-0.19	0.03 ± 0.03	0.07 ± 0.08	-0.52
Age at onset, years	31.1 ± 19.0	38.7 ± 19.0	-0.40	33.1 ± 19.9	37.9 ± 19.1	-0.25
Disease duration, months	205 ± 114	192 ± 156	0.09	183 ± 105	143 ± 115	0.36
Past relaplse, times	4.2 ± 3.3	2.7 ± 4.1	0.38	4.4 ± 3.5	1.5 ± 2.4	-0.93
past relapse per year, times/yr	0.29 ± 0.19	0.15 ± 0.19	0.72	0.33 ± 0.19	0.09 ± 0.13	1.45
Follow up period, months	44.6 ± 4.8	56.6 ± 10.2	-1.50	45.5 ± 4.7	51.6 ± 14.5	-0.57
Other immunosupressants, no./%	6, 46%	10, 27%	0.41	5, 45%	2, 18%	0.61
Comcomitant allergic disorders, no./%	13, 100%	20, 54%	1.30	11, 100%	5, 45%	1.55
Dyslipedemia, no./%	1, 8%	14, 38%	-0.77	1, 9%	5, 45%	-0.89
minimum PSL dose, mg	0.83 ± 1.1	2.6 ± 2.9	-0.37	0.93 ± 1.2	2.66 ± 3.86	-0.60
PSL dose at baseline, mg	4.1 ± 3.7	3.3 ± 3.7	0.21	3.8 ± 3.5	4.2 ± 4.5	-0.09
*IgE, U/mL	780 ± 643	496 ± 592	0.46	780 ± 643	653 ± 593	-0.21
Eosinophil, /microL	80 ± 62	224 ± 198	-0.98	80 ± 62	205 ± 133	-1.21
*Infection, irregular PSL reduction, or malignancy, no./%	4, 31%	16, 42%	-0.26	4, 36%	4, 36%	0.00

* matched items

Table S3 Characteristics of propensity score matched patients (model 2)

group	case	age at baseline	sex	Complicated allergy	immuno-suppressants	PSL dose		Minimum PSL dose		corticosteroid-free remission, months	IgE, U/mL	Infection, irregular corticosteroid reduction, or malignancy
						baseline, mg	after the study, mg (PSL dose on relapse, mg)	baseline, mg	after the study, mg			
Treatment (model 2)	1	62	F	AR	MZR	1	0	1	0	42	620	
	2	41	M	AD, As		1	relapse (0)	1	0	relapse (7)	12	
	3	45	M	AD, D	CyA	10	relapse (0.5)	0	0	relapse	1438	Bladder cancer, bronchiolitis
	4	30	M	AR, F	CyA	2	0	0	0	33	746	
	5	51	F	AD,S		0.5	0	0	0	37	182	
	6	38	M	AR		2	0	1	0	29	721	
	8	39	F	AD, AR, D, F	Tac	10	relapse (0)	0	0	relapse (13)	33	Herpes zoster virus, influenza
	10	32	F	AD, AR,S		3	0	2	0	37	1366	
	11	82	F	AD, D		8	0	4	0	37	93	Herpes zoster virus
	12	43	M	AD, AR		1.66	0	1.33	0	24	1937	
	13	65	F	AR	CyA	3	0	0	0	41	1430	Rectum adenocarcinoma
Control (model 2)	17	76	M	As	CyA	0	0	0	0	60	1190	pyelonephritis
	19	47	F	AD		0	0	0	0	60	1050	

23	19	M	AR		10	relapse (5)	0	0	relapse	1650	Influenza
25	43	M	U		3	2	0	0	on PSL	195	
27	76	F			0	0	0	0	60	351	
28	67	M			0	0	0	0	60	1638	
29	48	M		MZR	1.25	relapse (1.25)	1.25	1.25	relapse (0)	336	
30	28	F			10	0	10	0	6	23	
31	64	M			10	0	10	0	29	593	
32	29	F			10	0	6	0	38	107	Viral bronchiolitis
33	50	M	AR, D		2	relapse (2)	2	2	relapse (0)	50	Viral bronchiolitis

Table S4 Coefficient of Univariate logistic regression analysis of relapse during observation for patients without anti-allergy therapy

Variable	Odds ratio	Lower 95 % CI	Upper 95% CI	p value
Past relapse per year, 1 time per year	96.4	1.6	6000	0.030
Immunosupressant, yes	6.6	1.3	32.5	0.020
Age, 10 years	0.95	0.91	1.00	0.050
Disease duration, 1 year	1.00	0.99	1.00	0.30
Urinary protein, 1 g/gCre	0.25	0.00	45.5	0.60
eGFR, 1 ml/min/1.73m2	1.00	0.96	1.04	0.93
Minimum PSL dose, 1 mg	1.00	0.79	1.27	0.99
Concomitant allergic disorders, yes	1.09	0.25	4.71	0.90
PSL dose at baseline, 1 mg	1.09	0.91	1.32	0.34
Dyslipedemia, yes	0.51	0.11	2.38	0.39
Hyperurecemia, yes	0.77	0.07	8.30	0.83
Hypertension, yes	1.03	0.25	4.24	0.97
Diabetes mellitus, yes	2.26	0.52	9.83	0.28
Infection, irregular corticosteroid reduction, or malignancy, yes	1.70	0.24	12.00	0.59
IgE, 1 U/mL	1.00	1.00	1.00	0.29
Eosinophil, 1 /microg	1.00	1.00	1.00	0.65

Table S5. Characteristics of patients with or without allergy disorders at baseline

	allergy		
	no	yes	p
n	17	34	
male / female	10 / 7	22 / 12	0.757
albumin, g/dL	1.7 [1.2-2.7]	1.8 [1.2 - 2.3]	0.797
TP, g/dL	4.4 [4.0-5.4]	4.8 [4.0-5.3]	0.951
IgE, U/mL	179 [95-340]	620 [149-1278]	0.123
IgG, mg/dL	541 [390-1004]	537 [413-739]	0.682
IgA, mg/dL	270 [173-360]	288 [220-362]	0.515
IgM, mg/dL	128 [105-176]	157 [105-216]	0.227
Eosinophil, cells/microL	170 [104-267]	138 [43-884]	0.478
eGFR, ml/min/1.73m2	67 [61-72]	80 [72-92]	0.001
Urinary protein, g/gCre	0.04 [0.03-0.06]	0.03 [0.02-0.05]	0.318
Disease duration, months	165 [40-307]	159 [107-270]	0.790
Age, years	65 [53-73]	44 [40-59]	0.011
Past relaplse, times	1 [0-2]	3 [1-5]	0.008
Past relapse per year, times/yr	0.031 [0.0-0.084]	0.178 [0.086-0.398]	0.001
Follow up period, months	60 [60-60]	60 [56-60]	0.024
Other immunosupressants, no./%	2, 11.8%	14, 41.2%	0.053
Cyclosporin, no./%	1, 5.9%	12, 35.3%	0.038
Bredinine, no./%	1, 5.9%	2, 5.9%	1.000
Tacrolimus, no./%	0, 0%	1, 2.9%	1.000
Medication required comcomitant disease, no./%	12, 70.6%	21, 61.8%	0.757
Hypertention, no./%	7, 41.2%	19, 55.9%	0.373

Dyslipidemia, no./%	10, 58.8%	5, 14.7%	0.003
Diabetes mellitus, no./%	7, 41.2%	6, 17.6%	0.099
Hyperurecemia, no./%	1, 5.9%	6, 17.6%	0.398
Least PSL dose in disease history, mg	0 [0-1.25]	0 [0-1.5]	0.761
PSL dose at inclusion, mg	0 [0-3.125]	2.5 [1.5-8.0]	0.034
Cre at inclusion	0.83 [0.70-0.90]	0.80 [0.63-0.81]	0.219

Table S6. Coefficient of univariate logistic regression analysis of concomitant allergy disorders for all patients

Variable	Odds ratio	Lower 95 % CI	Upper 95% CI	p value
Past relapse per year, 1 time per year	836.0	2.2	320000	0.027
Immunosupressant, yes	3.8	0.7	19.7	0.109
Age, 10 years	0.97	0.94	1.01	0.164
Disease duration, 1 year	1.00	1.00	1.00	0.652
Urinary protein, 1 g/gCre	1.52	0.12	18.7	0.742
eGFR, 1 ml/min/1.73m2	1.02	0.99	1.06	0.197
Minimum PSL dose, 1 mg	0.90	0.72	1.12	0.336
Dyslipedemia, yes	0.29	0.08	1.06	0.061
Hyperurecemia, yes	2.60	0.28	23.80	0.398
Hypertension, yes	3.93	1.03	15.00	0.045
Diabetes mellitus, yes	0.51	0.13	1.98	0.333
Infection, irregular corticosteroid reduction, or malignancy	3.71	0.42	32.90	0.238
IgE, 1 U/mL	1.00	1.00	1.00	0.185
Eosinophil, 1 /microg	1.00	1.00	1.00	0.800

Supplementary material
Figure S1

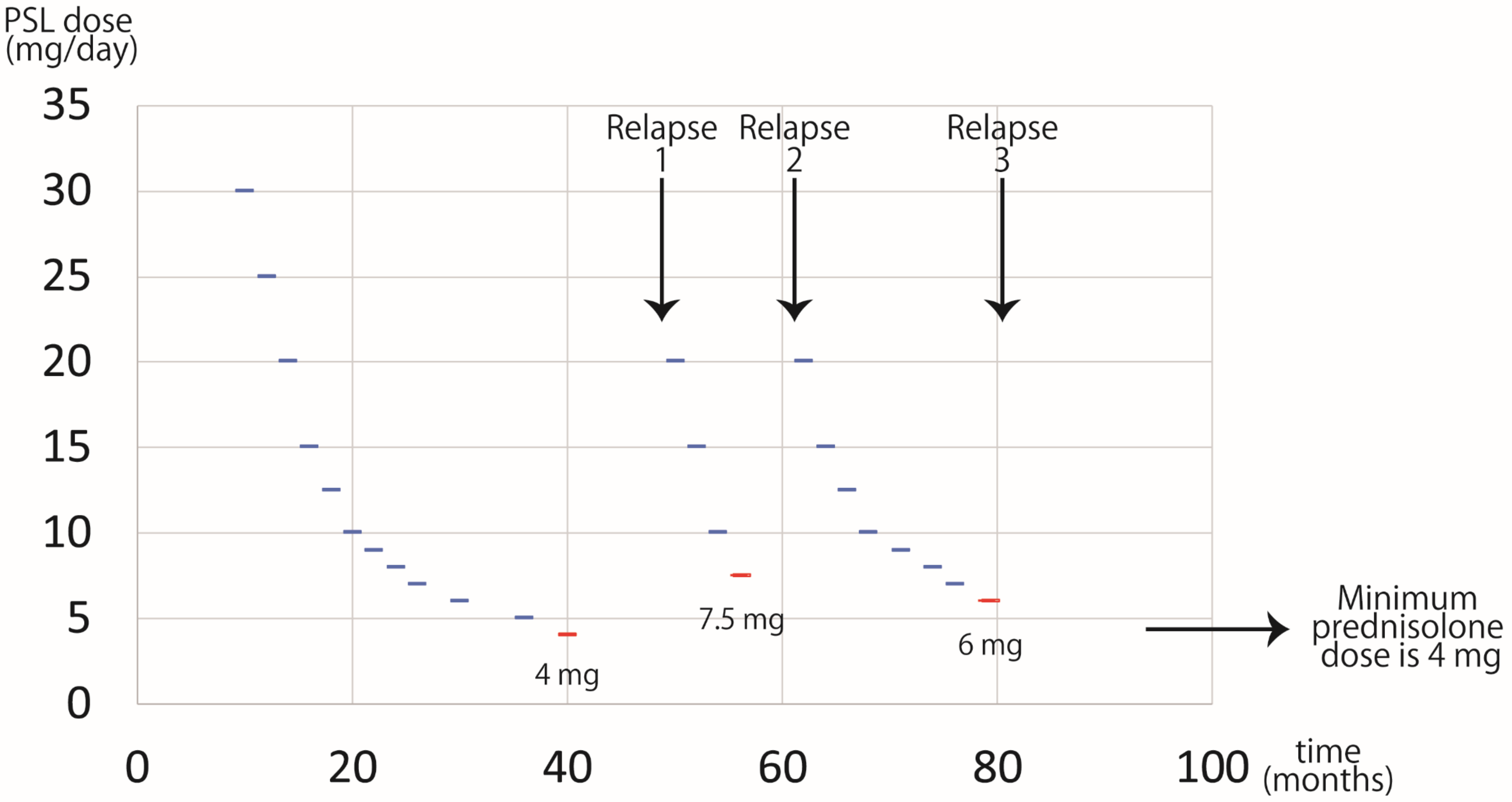

Supplementary figure
Figure S2

a

Successful reduction of minimum maintenance predonisolone dose after addition of cetirizine and montelukast in one patient

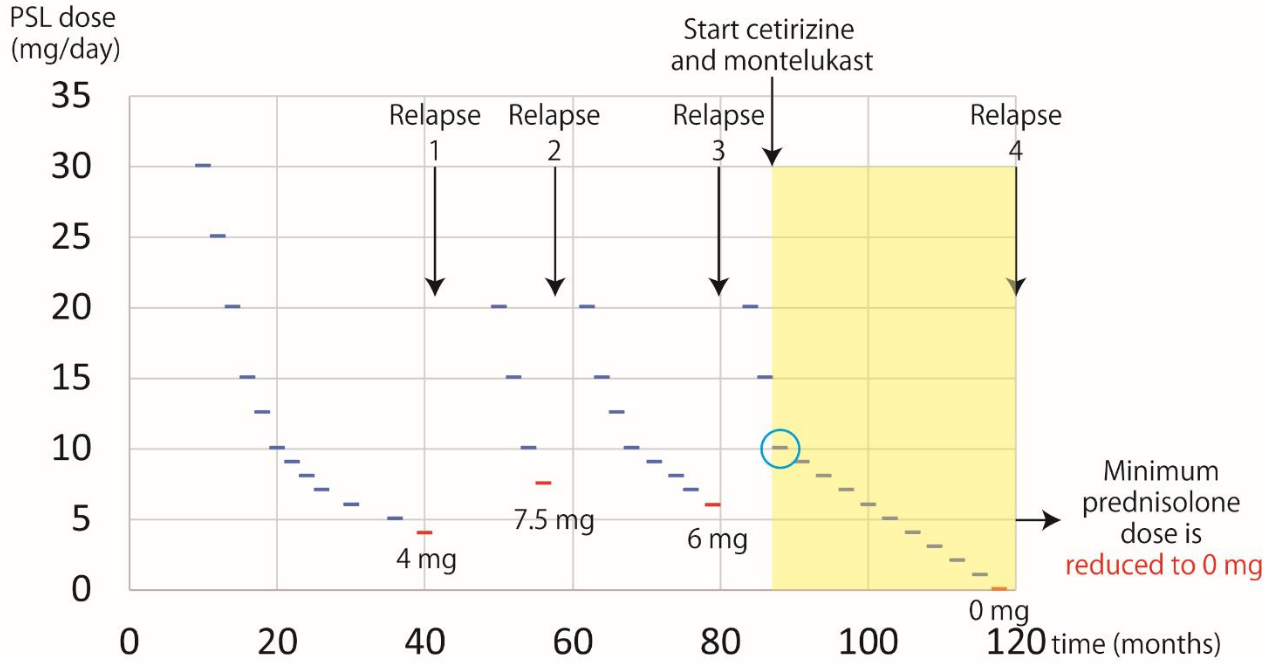

b

No reduction of minimum maintenance predonisolone dose

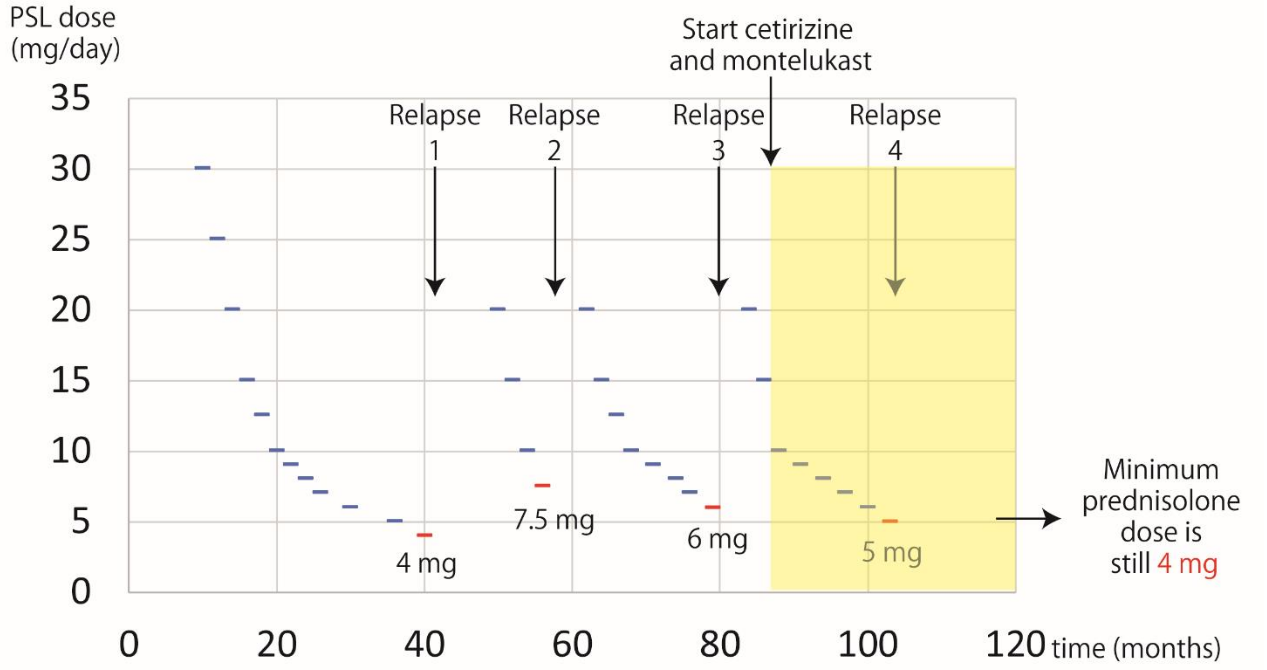

Figure S3

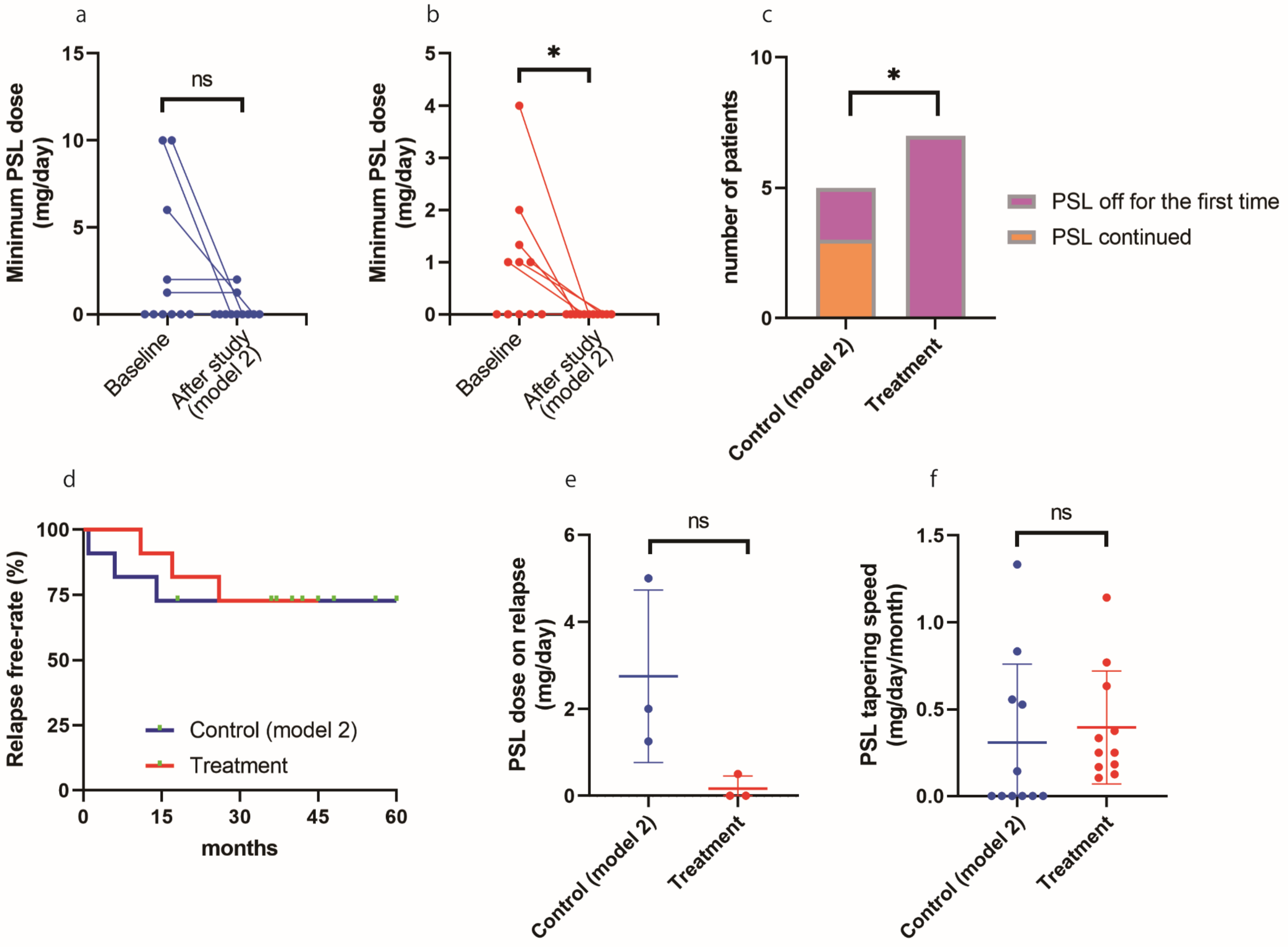
a
Minimum PSL dose (mg/day)
15
10
5
0
ns
Baseline
After study (model 2)
b
Minimum PSL dose (mg/day)
5
4
3
2
1
0
*
Baseline
After study (model 2)
c
number of patients
10
5
0
*
Control (model 2)
Treatment
PSL off for the first time
PSL continued
d
Relapse free-rate (%)
100
75
50
25
0
0
15
30
45
60
months
Control (model 2)
Treatment
e
PSL dose on relapse (mg/day)
6
4
2
0
ns
Control (model 2)
Treatment
f
PSL tapering speed (mg/day/month)
1.5
1.0
0.5
0.0
ns
Control (model 2)
Treatment
